# Developing a Pathway for Referrals of Patients With Dementia Within a Mental Health Liaison Team

**DOI:** 10.1192/bjo.2024.353

**Published:** 2024-08-01

**Authors:** Alex Burns, Nadia Imran

**Affiliations:** South West Yorkshire Partnership NHS Foundation Trust, Barnsley, United Kingdom

## Abstract

**Aims:**

Assessment and management of the mental health needs of patients with dementia has been identified as a key role for a mental health liaison team (MHLT). The existing practice for referrals of patients with dementia made to Barnsley Hospital's MHLT was for them to be redirected to the memory team for assessment, who have limited scope for in-reach work into hospital, rather than being assessed by MHLT who are based on the hospital site.

This project aimed to clarify the pathway for dementia referrals presenting with psychiatric issues at Barnsley Hospital and determine which patients should be seen by either MHLT or the memory team. It also aimed for MHLT to increase the number of dementia referrals assessed compared with existing practice and increase the proportion of face-to-face reviews for these patients.

**Methods:**

2 periods of data collection took place within MHLT, where the outcome of referrals made from Barnsley Hospital for patients with diagnosed or suspected dementia requiring assessment was recorded. The first period recorded existing practice and the second period recorded practice following the implementation of a new pathway for referrals.

The new referral pathway was created in collaboration between MHLT, memory team and Barnsley Hospital's dementia nursing staff. MHLT would review cases of suspected dementia not currently open to memory team whilst referrals made for patients open to memory team would be referred to memory team initially, with the option of MHLT input subsequently being requested.

**Results:**

First data collection period 3–28 April 2023:

4 referrals in total.

2 were assessed by MHLT, 1 seen face-to-face, 1 by telephone. 2 were redirected to memory team.

Second data collection period 17 July–17 September 2023 following implementation of the pathway:

10 referrals in total.

7 were assessed by MHLT, 7 seen face-to-face. 3 were redirected to memory team.

**Conclusion:**

The implementation of the pathway led to improved outcomes, with absolute increases of 20% in the proportion of referrals assessed by MHLT and of 45% in the proportion of patients assessed face-to-face. Undertaking the project also helped to identify that there was a training need for MHLT practitioners regarding dementia assessment and management. The next aim is for MHLT to assess 100% of dementia referrals following dementia training being delivered to the MHLT practitioners, and to continue regular MDT meetings to monitor the efficacy of the pathway and maintain collaboration between MHLT and the memory team.

